# Lactoferrin Deficiency Promotes Colitis-Associated Colorectal Dysplasia in Mice

**DOI:** 10.1371/journal.pone.0103298

**Published:** 2014-07-24

**Authors:** Qiurong Ye, Ying Zheng, Songqing Fan, Zailong Qin, Nan Li, Anliu Tang, Feiyan Ai, Xuemei Zhang, Yanhui Bian, Wei Dang, Jing Huang, Ming Zhou, Yanhong Zhou, Wei Xiong, Qun Yan, Jian Ma, Guiyuan Li

**Affiliations:** 1 Hunan Provincial Tumor Hospital and the Affiliated Tumor Hospital of Xiangya School of Medicine, Cancer Research Institute, Central South University, Hunan Key Laboratory of Nonresolving Inflammation and Cancer, Key Laboratory of Carcinogenesis, Ministry of Health, Key Laboratory of Carcinogenesis and Cancer Invasion, Ministry of Education, Changsha, Hunan, China; 2 Center for Medical Research, Second Xiangya Hospital, Central South University, Changsha, Hunan, China; 3 Department of Pathology, Second Xiangya Hospital, Central South University, Changsha, Hunan, China; 4 Department of Gastroenterology, Third Xiangya Hospital, Central South University, Changsha, Hunan, China; 5 Department of Laboratory Medicine, Xiangya Hospital, Central South University, Changsha, Hunan, China; Charité, Campus Benjamin Franklin, Germany

## Abstract

Nonresolving inflammatory processes affect all stages of carcinogenesis. Lactoferrin, a member of the transferrin family, is involved in the innate immune response and anti-inflammatory, anti-microbial, and anti-tumor activities. We previously found that lactoferrin is significantly down-regulated in specimens of nasopharyngeal carcinoma (NPC) and negatively associated with tumor progression, metastasis, and prognosis of patients with NPC. Additionally, *lactoferrin* expression levels are decreased in colorectal cancer as compared with normal tissue. Lactoferrin levels are also increased in the various phases of inflammation and dysplasia in an azoxymethane–dextran sulfate sodium (AOM-DSS) model of colitis-associated colon cancer (CAC). We thus hypothesized that the anti-inflammatory function of lactoferrin may contribute to its anti-tumor activity. Here we generated a new *Lactoferrin* knockout mouse model in which the mice are fertile, develop normally, and display no gross morphological abnormalities. We then challenged these mice with chemically induced intestinal inflammation to investigate the role of lactoferrin in inflammation and cancer development. Lactoferrin knockout mice demonstrated a great susceptibility to inflammation-induced colorectal dysplasia, and this characteristic may be related to inhibition of NF-κB and AKT/mTOR signaling as well as regulation of cell apoptosis and proliferation. Our results suggest that the protective roles of lactoferrin in colorectal mucosal immunity and inflammation**-**related malignant transformation, along with a deficiency in certain components of the innate immune system, may lead to serious consequences under conditions of inflammatory insult.

## Introduction

Lactoferrin (LF; or lactotransferrin, LTF) is a protein involved in a large variety of activities in mammals, all of which provide protective effects (anti-microbial, anti-inflammatory, and immune modulatory) for the host [1], [2]. Lactoferrin is abundant in the secondary granules of neutrophils; in biological fluids such as milk, tears, saliva, seminal plasma; and also in the secretions of nasal, pancreatic, gastrointestinal, bronchial, and uterine tissues [3]. Additionally, lactoferrin has a suppressive function in a variety of tumors [4]–[9]. In our previous study, we found that lactoferrin is significantly down-regulated in specimens of nasopharyngeal carcinoma (NPC) and, in patients with NPC, is negatively associated with tumor progression, metastasis, and prognosis. Lactoferrin inhibits NPC cell proliferation, induces cell cycle arrest at G1/S phase, and inhibits both MAPK and AKT signaling [10]–[12].

The exact mechanism by which lactoferrin inhibits cancer development is unclear. We hypothesized that such activity might be related to its anti-inflammatory function. Inflammatory responses play important roles at different stages of tumor development including initiation, promotion, invasion, and metastasis. During inflammation, levels of lactoferrin in biological fluids and epithelial cells increase dramatically [13]. Unlike many other molecular entities associated with inflammatory responses, lactoferrin displays a modulatory role by up-regulating or down-regulating inflammatory responses, depending on the status of the host and the inflammation environment (reviewed by Legrand and Mazurier [14]).

Most studies concerning lactoferrin’s anti-inflammatory response have been conducted *in vitro*. There is, however, a *Lactoferrin* knockout mouse model that was generated by Ward et al. [15], [16]. Those authors reported that no overt phenotypic abnormalities are found in *Lactoferrin* knockout mice maintained under normal physiological conditions, which implies that lactoferrin functions redundantly with other molecules such as transferrin *in vivo*.

Colitis-associated colon cancer (CAC), which arises as a result of an ‘inflammation-dysplasia-carcinoma’ sequence has been widely studied as a model for the link between inflammation and cancer. The combination of azoxymethane (AOM), a colonic genotoxic carcinogen, and dextran sulfate sodium (DSS), an inducer of colitis, has been frequently used to generate a mouse model of CAC. In the current study, we generated a new *Lactoferrin* knockout mouse model and challenged it with AOM-DSS–induced intestinal inflammation to examine the role of lactoferrin in the link between inflammation and cancer *in vivo*.

## Materials and Methods

### Generation of *Lactoferrin* knockout mice

**Figure 1:**
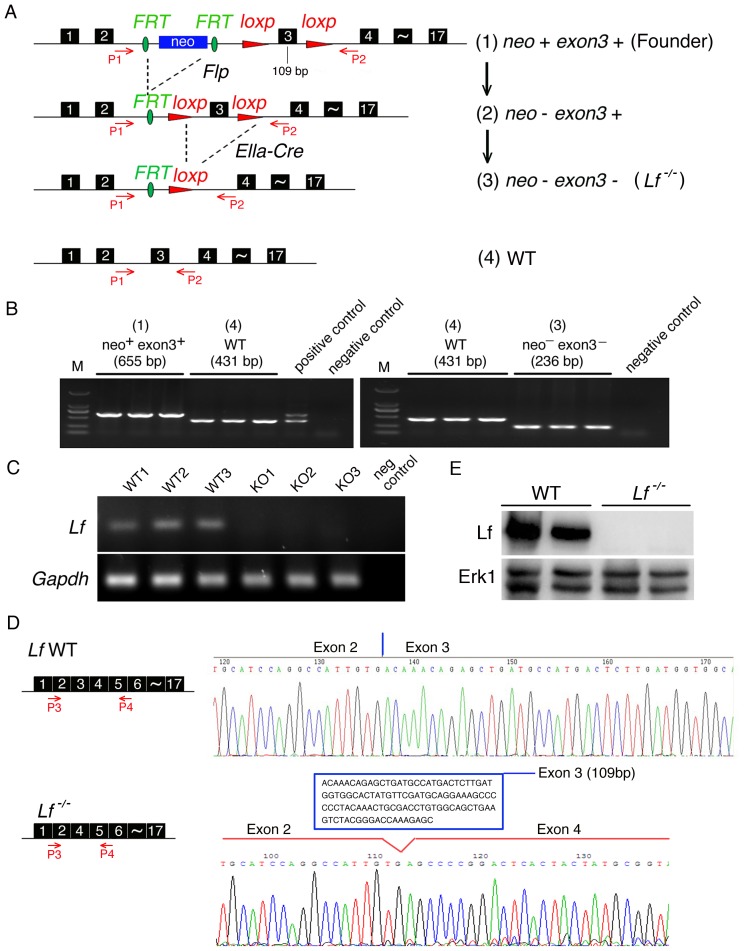
Generation of *Lactoferrin* (*Lf*) knockout mice. (A) Schematic diagram of the strategy used to generate *Lactoferrin* knockout mice. (1) Founder mice: Exon 3 of mouse *Lf* is flanked by *loxp* sequences. *FRT*-flanked *neo* is located between exon 2 and exon 3. (2) The *neo* cassette is deleted by crossing the founder mice with transgenic *Flp* mice. (3) Exon 3 is deleted by crossing the *neo* ^–^ *exon 3* ^+^ mice with transgenic *EIIa*-*Cre* mice. (4) Wild-type *Lf*, which is also generated from the same cross as in (3), has 17 exons. P1 and P2 indicate primers used for PCR analysis. (B) Genotyping of founder mice (1), WT mice (4), and *Lf* knockout mice (3) by PCR with primers P1 and P2. (C) RNA was harvested from the tails of three *Lactoferrin* knockout mice (KO1–KO3) and three wild-type (WT1–WT3) mice. RT-PCR with primers Lf1 and Lf2 was used to confirm that exon 3 was deleted from the KO mice. (D) Schematic diagram of RT-PCR primers P3 and P4 (left panel). The RT-PCR products from WT and *Lf* ^−/−^ mice were sequenced (right panel). Exon 3 was deleted from *Lf* ^−/−^ mice but not the wild type mice. (E) Western blot analysis was performed on mice bone marrow samples by using anti-mLf polyclonal antibody. Erk1 was used as loading control. All primers used in Figure 1 are listed in Table S1.

To obtain *Lactoferrin* knockout (*Lf* ^−/−^) mice that allowed both conditional and global disruption of the *Lf* gene, the *Cre/loxp* and *Flp/FRT* recombination systems were used to target exon 3 of *Lf*. Exon 3 contains 109 nucleotides, and its removal results in a frameshift mutation that splices exon 2 and exon 4. A single *loxp* site was inserted before exon 3 and before exon 4 to allow targeting of the pBR322 vector. A *FRT*-flanked PGK *neo* cassette was inserted into intron 2 before the *loxp* site to serve as a positive selectable marker (Figure 1A). The targeting vector was linearized with *Not I* and electroporated into mouse embryonic stem cells from 129 mouse strain. Germline *Lf^loxp-neo^* chimeras were obtained by homologous recombination. After obtaining mice that were homozygous for genotype *Lf^loxp-neo^,* the *FRT*-flanked PGK *neo* cassette was then deleted by crossing female *Lf^loxp-neo^* mice with transgenic *Flp* male mice to obtain mice that were homozygous for the *Lf^flox/flox^ Neo^–^* genotype. Mice with ubiquitous knockout of *Lf* (i.e. *Lf* ^−/−^) were generated in three steps. (1) *Lf* ^+/−^,*Cre* ^+/−^ mice were generated by crossing female mice homozygous for a floxed *Lf* allele (*Lf ^flox/flox^*) with transgenic EIIα *Cre* male mice from the Jackson Laboratory (Jackson, ME, USA), which carry a Cre recombinase (Cre expression is controlled by the EIIα promoter). (2) *Lf* ^+/−^,*Cre* ^−/−^ mice were generated by crossing the male *Lf* ^+/−^,*Cre* ^+/−^ mice with female C57BL/6J mice to delete the EIIα-*Cre* gene. (3) *Lf* ^+/−^,*Cre* ^−/−^ mice (heterozygotes) were intercrossed to obtain *Lf* ^−/−^ (homozygotes) and wild-type (WT) control mice for future experiments (Figure 1A). Genomic DNA was extracted from tail or ear biopsies and used as a template for PCR analysis with the appropriate primers to identify mouse genotypes (primers are listed in Table S1).

This study was carried out in strict accordance with the recommendations in the Guide for the Care and Use of Laboratory Animals (National Institutes of Health). The protocol was approved by the Animal Ethics Committee of Central South University. All surgeries were performed under sodium pentobarbital anesthesia, and all efforts were made to minimize animal suffering.

### Murine inflammatory carcinogenesis protocol

On day 1 of the study, male *Lf* ^−/−^ mice (n = 26; aged 6–8 weeks) and their male wild-type littermates (n = 13) were intraperitoneally injected with a single dose of 4 mg/kg body weight of AOM (Sigma-Aldrich, St. Louis, MO, USA) dissolved in physiological saline. Seven days after AOM administration, 1% DSS (MP Biomedicals, Solon, OH, USA; MW, 36–50 kDa) was administered through the drinking water for 7 consecutive days, followed by 14 days of distilled water. These 21 days constituted one cycle, and a total of three cycles were employed, and then distilled water was administered until the end of the experiment on day 126. These mice were sacrificed at the indicated time intervals for macroscopic inspection, total RNA and protein extraction, and histological analysis.

### Sample preparation

After the 18-week treatment period, all mice were sacrificed. The large bowel of each mouse was removed, cut open longitudinally along the main axis, and flushed with saline. The masses of inflamed and normal colonic mucosa were removed, immediately placed in RNAlater solution (Ambion, Austin, TX, USA), and stored in liquid nitrogen for subsequent RNA and protein extraction. The remaining section of each large bowel was fixed as a ‘Swiss roll’ in 4% paraformaldehyde overnight. Paraffin-embedded sections (4 µm thick) of the large bowel were then prepared by routine procedures for histopathological analysis and immunohistochemistry.

### Quantitative real-time PCR analysis (qPCR)

Total RNA was isolated using the RNeasy Mini kit (Qiagen, Valencia, CA, USA). cDNA was synthesized from the total RNA with a Reverse Transcription System (Fermentas, Glen Burnie, MD, USA). *Gapdh* was amplified in parallel as an internal control. The expression of each gene was quantified by measuring cycle threshold (Ct) values and normalized relative to *Gapdh* using the 2^−ΔΔCt^ method [17]. PCR primers are listed in Table S2.

### Histopathological analysis and immunohistochemistry

Histopathological analysis and immunohistochemistry were carried out as described [18]. Anti-p65 (Santa Cruz Biotechnology, Santa Cruz, CA, USA; 1∶200), anti-p-IKKα/β (Cell Signaling Technology, Danvers, MA, USA; 1∶100), anti-p-S6(S235/236) (Cell Signaling Technology; 1∶400), anti-AKT (Abcam, Cambridge, MA, USA; 1∶100), anti-p-AKT(S473) (Abcam; 1∶100); anti-Bax (Epitomics, Burlingame, CA, USA; 1∶300), anti-Ki67 (Bioss, Beijing, China; 1∶50), anti-IL-1β (Abzoom, Dallas, USA; 1∶200), anti-IL-6 (Sanying, Wuhan, China; 1∶300), and anti-TNF-α (Boster, Wuhan, China, 1∶150) were used in the immunohistochemical studies. Secondary antibody was diluted 1∶100 and applied for 2 h at room temperature. The Vectastain ABC Elite System (Vector Laboratories, Burlingame, CA) was used to visualize staining for IHC. The immunostained slides were observed under a microscope. Images were taken by a digital camera and semi-quantity of the signal was analyzed by counting mean density of the immunoreactivities against all primary antibodies. Brown or yellow was regarded as positive signal. Image data were analyzed with NIS-Elements AR 3.0 software (Nikon, Japan). Immune cell, apoptosis, and proliferation indices were generated by counting the number of positive cells per high-powered field (HPF; 40× objective) within each mouse [19].

### TUNEL assay

TUNEL assays were carried out on the sectioned tissue samples using the DeadEnd Colorimetric TUNEL System (Promega, Madison, WI, USA) as the manufacturer’s instructions.

### Western blotting

Large bowel tissues were homogenized and sonicated in RIPA lysis buffer (Beyotime Biotechnology, Suzhou, China) supplemented with phosphatase inhibitors (Roche, Mannheim, Germany). Western blotting was carried out as described [11]. Anti-p-Akt (S473) (Abcam), anti-p-S6 (S235/236) (Cell Signaling Technology), anti-Gapdh (Millipore, Billerica, MA, USA), anti-mLf polyclonal antibody (GenScript, Nanjing, China), anti-Erk1 (Santa Cruz Biotechnology) and anti-α-tubulin (Santa Cruz Biotechnology) were used. After centrifugation at 20,000 g for 15 min, 50 µg of the supernatants were separated by SDS-PAGE and transferred onto nitrocellulose membranes (Hyclone Laboratories). The membranes were blocked with 5% nonfat milk in Tris-buffered saline/Tween 20 (25 mM Tris-HCl, 150 mM NaCl, pH 7.5 and 0.05% Tween 20) and probed with primary antibody overnight at 4°C. After washing with Tris-buffered saline/Tween 20, the membranes were incubated with horseradish peroxidase-conjugated secondary antibodies (Santa Cruz Biotechnology) and visualized using the ECL detection system.

### Statistics

Fisher’s exact test (two tailed) was used to compare differences in dysplasia rates between *Lf* ^−/−^ and WT mice. The paired *t*-test was used to compare differences in *Lactoferrin* expression between CRC and non-tumor colon tissue. The Mann-Whitney test was used to compare relative differences in *Lactoferrin* expression between groups. Unless otherwise indicated, two-sample comparisons were made using the two-tailed Student’s unpaired *t*-test with pooled variance, if there was no evidence of non-homogeneity of variances between groups. The chi-square test was used to compare the positive ratio of the expressions of indicted molecules by immunohistochemistry between *Lf* ^−/−^ and WT mice [20]. P-values of <0.05 were considered statistically significant for all comparisons.

## Results

### Generation of *Lactoferrin* knockout (*Lf* ^−/−^) mice

Mice deficient in *Lactoferrin* (*Lf*) were generated by homologous gene targeting in embryonic stem cells. In this procedure, *Cre/loxp* and *Flp/FRT* recombination systems were used to target exon 3 of the mouse *Lf* gene (Figure 1A). Routine genotyping of mice was carried out by PCR amplification (Figure 1B and C). Sequencing of the PCR products showed that exon 3 of the *Lf* gene was successfully removed (Figure 1D). To confirm that we had successfully abolished the expression of the lactoferrin protein, we performed Western blot analysis on bone marrow samples by using anti-mLf polyclonal antibody (Figure 1E). A single immunoreactive band migrating at the size expected for lactoferrin protein was detected in the bone marrow from WT mice while no lactoferrin protein was detected in the bone marrow of *Lf* ^−/−^ mice. Based on the above procedures, the *Lf* ^−/−^ mice were successfully generated. The male and female *Lf* ^−/−^ mice were fertile, developed normally, and displayed no gross morphological abnormalities.

### *Lactoferrin* knockout mice showed higher susceptibility to AOM-DSS–induced colorectal dysplasia

**Figure 2:**
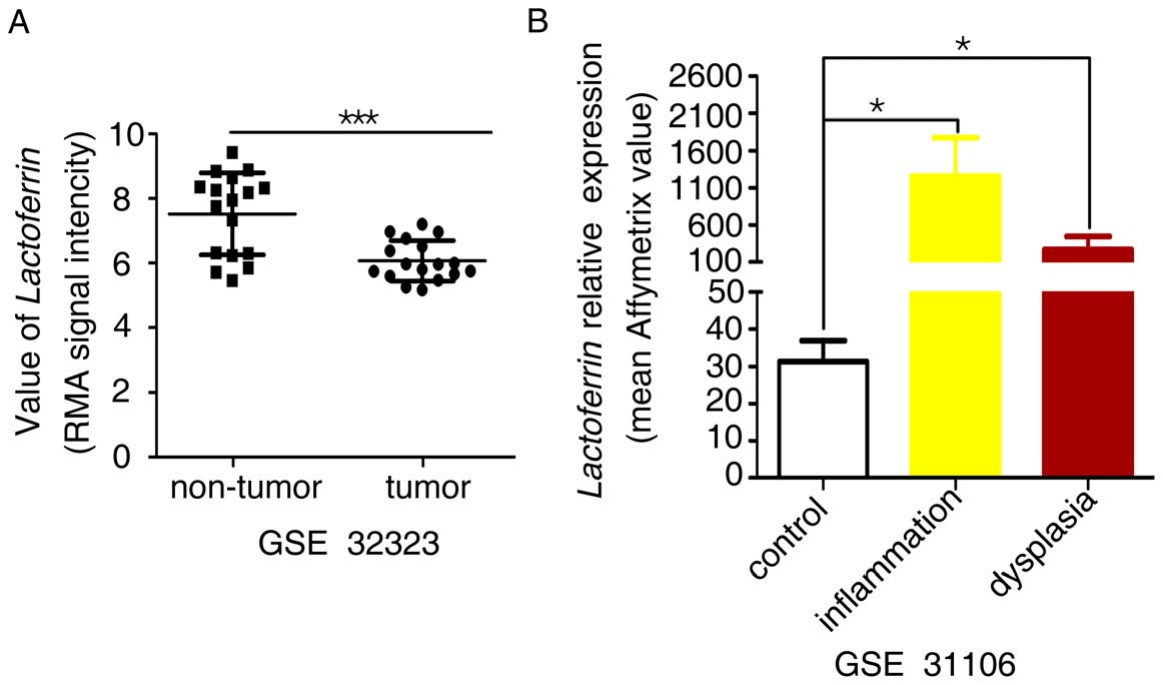
*Lactoferrin* gene expression levels in colorectal tissues. (A) *Lactoferrin* expression (RMA (robust multichip analysis) signal intensity) is decreased in human CRC tumor tissues as compared with non-tumor control tissues (data from NCBI GEO profiling dataset GSE 32323), P<0.001, paired test. (B) *Lactoferrin* gene expression is increased in tissues showing inflammation or dysplasia versus normal control tissues (data from NCBI GEO profiling dataset GSE 31106), P<0.05, Mann-Whitney test. Error bars represent the standard error.

AOM-DSS–induced colitis was introduced into *Lf* knockout mice to investigate the role of lactoferrin in the link between inflammation and cancer *in vivo.* This was done for several reasons. First, Ward et al [15] had previously reported that no overt phenotypic abnormalities were observed in *Lactoferrin* knockout mice maintained under normal physiological conditions. Second, we compared the *Lactoferrin* expression levels in human colorectal cancer (CRC) and non-tumor colon tissues using an mRNA expression profiling dataset [21] and found that *Lactoferrin* was significantly down-regulated in CRC tissue as compared with non-tumor tissues (Figure 2A). Third, we examined comprehensive gene expression data [18] (GSE31106) for murine colitis-associated cancer (CAC) tissues, which included pure inflamed CAC lesions of different pathological grades that were obtained from the AOM-DSS model. These lesions showed sharp increases in *lactoferrin* levels in tissues with inflammation or dysplasia (Figure 2B). All of the above data suggested a possible role for lactoferrin in the colitis-dysplasia-cancer linkage.

**Figure 3:**
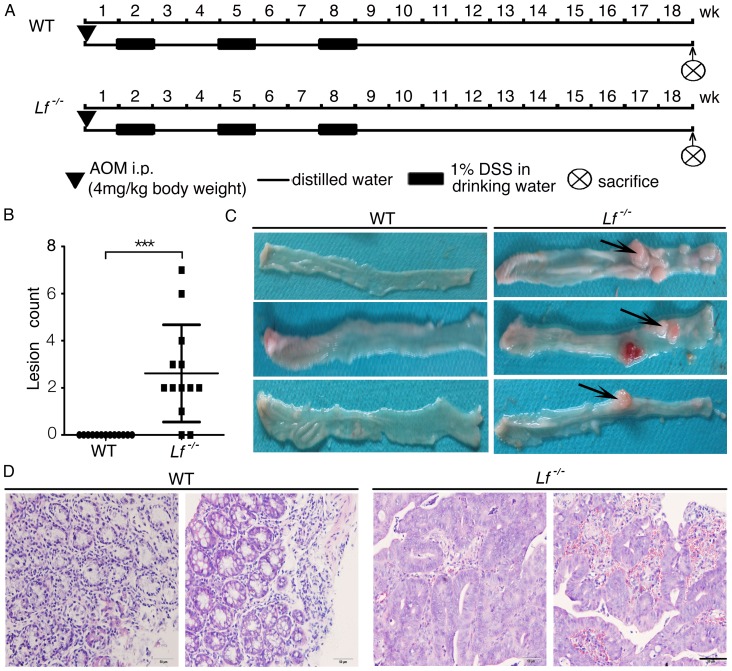
*Lactoferrin* knockout mice showed greater susceptibility to AOM-DSS–induced colorectal dysplasia. (A) Experimental procedure used for the AOM-DSS mouse model. (B) Intestinal lesion frequency as determined by calculating the lesion numbers of the intestinal surface area of each mouse. Each value represents the mean ± SD (n = 13/group), ***P<0.001. (C) Macroscopic changes in the colorectal tissues, black arrows indicate lesion. (D) Representative histopathological examination of *Lf* ^−/−^ and WT mice colorectal tissues (H&E staining, bar = 50 µm).

After two pilot experiments (data not shown), we chose a lower dose of AOM (4 mg/kg body weight) and DSS (1%) in the *lactoferrin* knockout mice (C57BL/6J background) compared with the doses selected for our previous procedure using ICR mice [18]. Male WT and *Lf* ^−/−^ mice (aged 6–8 weeks) were placed on the AOM-DSS protocol for 18 weeks (Figure 3A). Macroscopic observations of the colons from all AOM-DSS–treated *Lf* ^−/−^ mice, which were sacrificed on day 126, showed lesions in the middle to distal regions of the colon (Figure 3B, C). Histologic examination of H&E-stained sections of colons prepared as ‘Swiss rolls’ revealed the frequent occurrence of dysplasia characterized by loss of epithelial polarity in *Lf* ^−/−^ mice (Figure 3D). The rates of dysplasia were 31% in *Lf* ^−/−^ mice (8/26) and 0% in WT mice (0/13) (P = 0.0352, Fisher’s exact test, two tailed; Table 1), suggesting that *lactoferrin* deficiency promotes colitis-associated colorectal dysplasia.

**Table 1:** Incidence of colorectal dysplasia in the AOM-DSS mouse model.

Genotype	No. of mice examined	No. of mice with colorectal dysplasia	*P* value (Fisher’s exact test, two tailed)
Wild type	13	0	0.0352
*Lf* ^−/−^	26	8	

### The canonical NF-κB pathway was activated in *Lf* ^−/−^ mice

**Figure 4:**
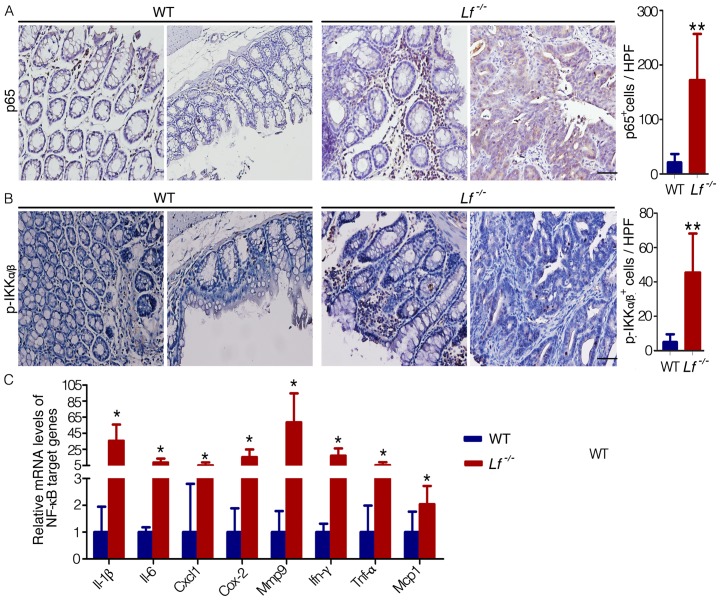
The canonical NF- κ**B pathway was activated in** ***Lf*** **^−/−^ mice.** The colons of AOM-DSS–treated mice were removed after 18 weeks. (A,B) Immunohistochemical analysis of nuclear p65 (A) and cytoplasmic p-IKKα/β (B) expression in the colon tissues. Cells that stained positive for p65 or for cytoplasmic p-IKKα/β were counted per high-powered field (HPF, 40× objective, bar = 50 µm). (C) mRNA expression levels of *Il-1β, Il-6, Cxcl1, Cox-2, Mmp9, Ifn-γ, Tnf-α,* and *Mcp1* in the murine colons were assayed by qPCR. Each value represents the mean ± SD (n = 5 mice/group). The expression values for WT mice were set at 1 for each gene. *P<0.05, **P<0.01 versus WT mice.

The anti-inflammatory role of lactoferrin has been widely reported, and it has been suggested that lactoferrin suppresses activation of NF-κB signaling by multiple mechanisms. For instance, lactoferrin inhibits lipopolysaccharide-induced DNA-binding activity of NF-κB, as well as IκBα and IKKβ phosphorylation [22]. We hypothesized that the inhibition of colitis-associated colorectal dysplasia by lactoferrin in mice may be related to its anti-inflammatory function. We used immunohistochemistry to determine the levels of transcription factor p65 in the nucleus and of p-IKKα/β in the cytoplasm, which are both markers of NF-κB activation [23]. Nuclear p65 protein expression levels and p65^+^ cell numbers were increased in *Lf* ^−/−^ mice compared with WT mice, and similar results were found for p-IKKα/β (Figures 4A, B). The mRNA expression levels of certain NF-κB transcription target genes (including *Il-1β, Il-6, Cxcl1, Cox-2, Mmp9, Ifn-γ, Tnf-α* and *Mcp1*) were significantly increased in the colon tissues of *Lf* ^−/−^ mice compared with the colon tissues of wild-type mice (Figure 4C). These results clearly suggest that in an inflammatory environment, lactoferrin suppresses NF-κB signaling *in vivo*.

### Lactoferrin induced apoptosis and inhibited cell proliferation in the AOM-DSS model

**Figure 5:**
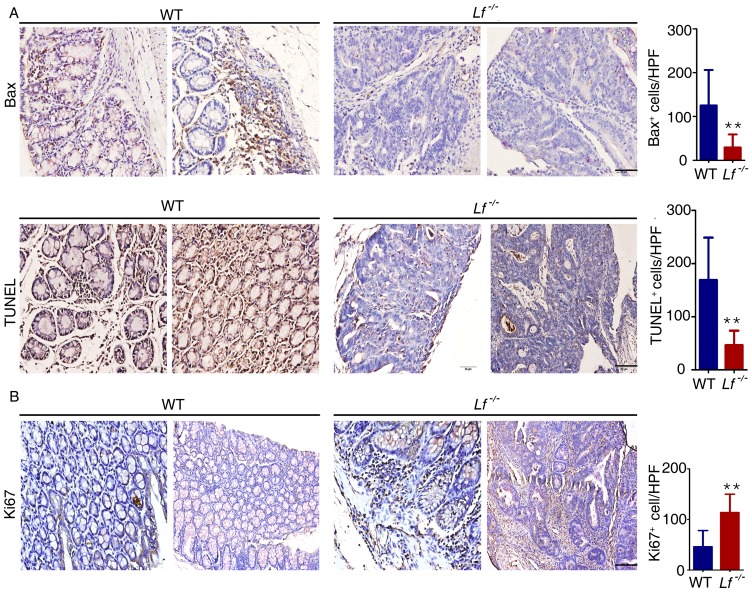
*Lf* ^−/−^ mice demonstrated decreased apoptosis and increased proliferation. The colons of AOM-DSS–treated mice were removed after 18 weeks. (A) (upper) Immunohistochemistry analysis of Bax protein in the colon tissues. (lower) Apoptosis was measured via TUNEL staining of ‘Swiss roll’ colons from mice. (B) Immunohistochemistry analysis of Ki67 expression. Scale bar is 50 µm. Cells that stained positive were counted per HPF (40× objective). **P<0.01 versus WT mice. Each value represents the mean ± SD (n = 10 mice/group).

Normal tissues carefully maintain a balance between cell proliferation and apoptosis, thus ensuring homeostasis of cell numbers and the maintenance of normal tissue architecture and function. However, under conditions of inflammation or disease, this balance can be disrupted. Lactoferrin promotes cell apoptosis by activating both extrinsic and intrinsic apoptotic pathways [24]. We used immunohistochemistry to determine that the level of the pro-apoptotic protein Bax in mouse colon tissues was decreased in *Lf* ^−/−^ mice compared with expression in WT mice (Figure 5A). Apoptotic signaling was assessed by TUNEL staining of the colon tissues. *Lf* ^−/−^ mice demonstrated an average of 46.67±27.33 TUNEL^+^ cells/high-powered field (HPF), whereas WT mice displayed 169.0±79.84 TUNEL^+^ cells/HPF (P<0.01, *Lf* ^−/−^ versus WT; Figure 5A).

Lactoferrin inhibits the proliferation of various types of cells by altering the levels of certain crucial regulators [5], [25], [26]. Ki67, a nuclear antigen expressed during all stages of the cell cycle except G0, is the proliferation marker [27]. Ki67 immunohistochemistry was performed to analyze the cellular proliferation rates. The number of Ki67^+^ cells/HPF in *Lf* ^−/−^ mice was much higher compared with the number in WT mice (P<0.01; Figure 5B). Overall, *Lf* ^−/−^ mice demonstrated lower rates of apoptosis and higher proliferation indexes compared with WT mice in the AOM-DSS model.

### The AKT/mTOR pathway was activated in *Lf* ^−/−^ mice

**Figure 6:**
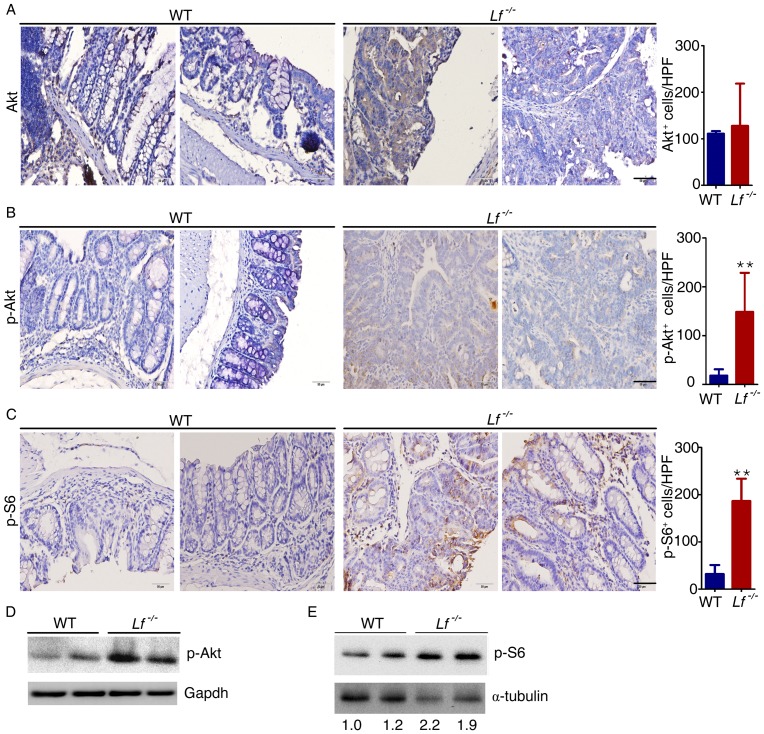
The AKT/mTOR pathway was activated in *Lf* ^−/−^ mice. The colons were removed from AOM-DSS–treated mice after 18 weeks. (A–C) Immunohistochemical analysis of AKT (A), p-AKT (B), and p-S6 (C) expression levels in colon tissues. Cells that stained positive were counted per HPF (40× objective). **P<0.01 versus WT mice. Scale bar is 50 µm. Each value represents the mean ± SD (n = 10 mice/group). (D,E) Western blot analysis of p-AKT (D) and p-S6 (E) expression levels in the colon tissues.

AKT kinase is involved in the regulation of various signaling pathways, including those involved in metabolism, cell proliferation, survival, growth, and angiogenesis. The AKT kinase pathways are among the most important pathways for regulating cell proliferation [28]. We previously reported that LF suppresses NPC cell proliferation and invasiveness by inhibiting AKT signaling [12], and we therefore investigated whether activation of AKT signaling was affected in the *Lf* ^−/−^ mouse model. Akt was equally expressed in *Lf* ^−/−^ mice and WT mice (Figure 6A); however, p-Akt expression levels were significantly increased in the colon tissues of AOM-DSS–treated *Lf* ^−/−^ mice as compared with the colon tissues of WT mice (Figure 6B, D). The mTOR serine-threonine kinase is a central mediator of several signal transduction pathways and is considered a regulator of cell proliferation, protein translation, and survival. The phosphorylation of ribosomal protein S6 (p-S6) is widely used as an indicator of mTOR activity in human tumor specimens. In this study, p-S6 levels were upregulated in the colon tissues of AOM-DSS–treated *Lf* ^−/−^ mice compared with the colon tissues of WT mice (Figures 6C, E). These data suggest that lactoferrin is a negative regulator of AKT/mTOR signaling in this mouse model of inflammation. As previously reported, cytokines (such as IL-1β, IL-6, TNF-α, etc) abnormalities can lead to AKT activation and mTOR deregulation, and these cytokines are important mediators of inflammation related carcinogenesis. Therefore we assayed the association between cytokines and AKT/mTOR signaling using the immunohistochemistry of serial sections. Compared to the WT mice, the level of p-AKT and IL-1β were obviously increased in the same position of *Lf* ^−/−^ mice (Figure S1A), the same results were observed in p-S6, IL-6 and TNF-α (Figure S1B). These data suggest that the AKT activation and mTOR deregulation in the *Lactoferrin* deficiency mouse may be caused through multiple mechanisms including cytokines abnormalities.

## Discussion

To investigate the role of Lactoferrin *in vivo*, Ward *et al*. constructed the first *Lactoferrin* knockout mice and reported that no overt phenotypic abnormalities are observed under normal physiological conditions [15]. They also reported that lactoferrin is a positive modulator of the neutrophil oxidative burst in response to phorbol myristate-13-acetate (PMA) stimulation [16]. Using the same model, Velliyagounder’s group reported that *Lactoferrin* knockout mice have higher levels of alveolar bone loss, with increased expression of proinflammatory cytokines as well as chemokines during oral infection with *Aggregatibacter. actinomycetemcomitans(A. actinomycetemcomitans)* [29]. They also reported that administration of human lactoferrin could protect *Lactoferrin* knockout mice from *A. actinomycetemcomitans*–induced bacteremia [30].

We found that lactoferrin was significantly down-regulated in NPC [11] and CRC specimens as compared with non-tumor tissues (Figure 2A). There was also an increase in *lactoferrin* expression in the AOM-DSS–induced colitis-dysplasia mouse model (Figure 2B). We therefore hypothesized that lactoferrin’s anti-inflammatory function may contribute to its anti-tumor function.

In the current study, we established a new *Lactoferrin* knockout mouse model and found that the *Lf* ^−/−^ mice were fertile, showed normal development, and displayed no gross morphological abnormalities; these findings are similar to those reported by Ward et al. [15]. To investigate the role of lactoferrin in inflammation-cancer development, we chose the AOM-DSS–induced inflammation model in which to monitor the difference between *Lf* ^−/−^ and WT mice. Based on the background difference between ICR and C57BL/6J mice, we used relatively low doses of AOM (4 mg/kg) and DSS (1%) for this study. Our results showed that when using such low doses, the WT mice developed colitis, but not dysplasia or cancer, during the 18-week period (Figure 3D). In contrast, among the *Lf* ^−/−^ mice, 100% developed colitis, and 31% developed middle or high-grade dysplasia. High-grade dysplasia in mice is similar to human intestinal carcinoma *in situ* (or microcarcinoma), which is characterized by more-pronounced nuclear atypia, with loss of epithelial cell nuclear polarity [31]. Additionally, the neoplastic glands may show architectural complexities such as a cribriform appearance [31]. These observations suggest that lactoferrin plays a protective role in the process of cancer development that is induced by nonresolving inflammation.

Lactoferrin exerts anti-inflammatory effects by activating innate immune cells, decreasing the release of cytokines, reducing immune cell recruitment at inflammatory sites, and depriving microbes of iron ions [1], [32]. We demonstrated that both the nuclear staining of p65 and phosphorylation of IKKα/β were increased in *Lf* ^−/−^ mice and that expression of downstream target genes of NF-κB (including genes that encode inflammatory agents such as *Il-1β, Il-6, Cxcl1, Cox-2, Mmp9, Ifn-γ, Tnf-α,* and *Mcp1*) were significantly higher in *Lf* ^−/−^ mice compared with WT mice (Figure 4). The resulting increase in inflammatory agents not only aggravates existing inflammation but also promotes tumorigenesis. For instance, prolonged exposure to Il-1β, Mcp1, or Il-6 leads to the formation of an inflammatory microenvironment, which inhibits the repair of inflammatory lesions [31]. Release of Il-1β and Il-6 results in further activation and proliferation of epithelial cells by NF-κB and STAT3 in a MAPK-dependent manner [18]. In addition, inhibition of COX-2 can inhibit the progression of dysplasia to adenocarcinoma [33]. The chemokine (C-X-C motif) ligand 1(CXCL1) is co-regulated by the NF-κB and STAT3 signaling, and its overexpression in dysplastic phases suggested that it may function as a tumor initiator [18]. MMP9 promotes tumor migration, and inhibition of MMP9 can suppress tumor invasion and migration. TNF-α is involved in the regulation of a wide spectrum of biological processes including cell proliferation, differentiation, and apoptosis; blocking expression of TNF-α can reverse carcinoma progression [34].

Lactoferrin facilitates cell apoptosis by activating the Fas and JNK pathways [35], [36]. In addition, inflammation can induce cell apoptosis and necrosis. Whereas apoptosis can be beneficial for an organism, necrosis is almost always detrimental and can be fatal. Under inflammatory conditions, *Lactoferrin* knockout mice showed much lower rates of apoptosis compared with rates in WT mice, indicating an increase in necrosis; such an increase may elicit a more intense inflammatory response (Figue 1C). Lactoferrin inhibits proliferation of multiple types of cancer cells, including cervical, gastric, and breast cancer [5], [25], [26]. Deletion of *Lactoferrin* results in aggravated inflammation and increased secretion of proinflammatory cytokines, which may accelerate the proliferation of colorectal cells, leading to tumorigenesis. We previously reported that lactoferrin suppresses tumorigenesis by inhibiting the AKT pathway [12]. In the current study, we observed increased secretion of proinflammatory cytokines and activity of AKT as well as its downstream mTOR signaling pathway in the colon tissues of *Lactoferrin* knockout mice as compared with the colon tissues of WT mice. As we know, the cytokines [37] (such as IL-1β, IL-6, TNF-α, etc) abnormalities can cause AKT activation and mTOR deregulation, prolonged exposure to proinflammatory cytokine not only aggravates existing inflammation but also promotes tumorigenesis. Thus, the AKT and mTOR deregulation in the *Lactoferrin* deficiency mouse may be caused through multiple mechanisms including cytokines abnormalities. AKT blocks FOXO-mediated transcription of target genes that promote apoptosis, cell cycle arrest, and metabolic processes. AKT also inhibits Bax expression, which leads to decreased apoptosis of tumor cells and higher levels of cell proliferation. AKT activation can also stimulate cell proliferation by affecting multiple downstream targets that affect cell cycle regulation. For instance, AKT phosphorylates and inhibits p21 [38]. This is significant because p21 can interact with proliferating cell nuclear antigen (PCNA), a DNA polymerase accessory factor, and assist in regulating S-phase DNA replication and DNA damage repair. The mTOR signaling pathway integrates cues related to the stage of the cell cycle, energy status, and the presence of growth factors to control cell size and proliferation. Activation of AKT leads to phosphorylation of mTORC1 and subsequent increases in phosphorylation of p70 S6K and 4E-BP1. These events lead to the downstream phosphorylation of ribosomal protein S6 (S6), resulting in translational initiation and cell growth [39].

The anti-inflammatory function of lactoferrin has been widely studied, and the underlying mechanism appears to involve several different pathways. In the current study, which was conducted *in vivo* and used a chemically induced nonresolving model of colitis, we demonstrated that lactoferrin deficiency promotes colitis-associated colorectal dysplasia in mice. This finding links the anti-inflammatory and anti-tumor functions of lactoferrin and highlights the protective roles played by lactoferrin in mucosal immunity and malignant transformation. Thus, a deficiency in certain components of the innate immune system may lead to serious consequences under conditions of inflammatory insult.

## Supporting Information

Figure S1 **The association between the effect of cytokines abnormalities and AKT activation and mTOR deregulation.** The colons were removed from AOM-DSS–treated mice after 18 weeks. (A) Immunohistochemistry analysis of serial sections of p-AKT and IL-1β expression levels in colon tissues. (B) Immunohistochemistry analysis of serial sections of p-S6, IL-6 and TNF-α expression levels in colon tissues. Cells that stained positive were counted per HPF (40× objective). *P<0.05, **P<0.01 versus WT mice. Scale bar is 50 µm. Each value represents the mean ± SD (n = 10 mice/group).
(JPG)

Table S1 **Primers used for identification of mouse genotypes by PCR.**
(PDF)

Table S2 **Primers used for detection of NF-κB target genes by qPCR.**
(PDF)
