# Supplementary figures and images for: Lactoferrin Deficiency Promotes Colitis-Associated Colorectal Dysplasia in Mice
Source: PLoS One. 2014 Jul 24;9(7):e103298. doi: 10.1371/journal.pone.0103298 (PMC4110006; doi:10.1371/journal.pone.0103298)

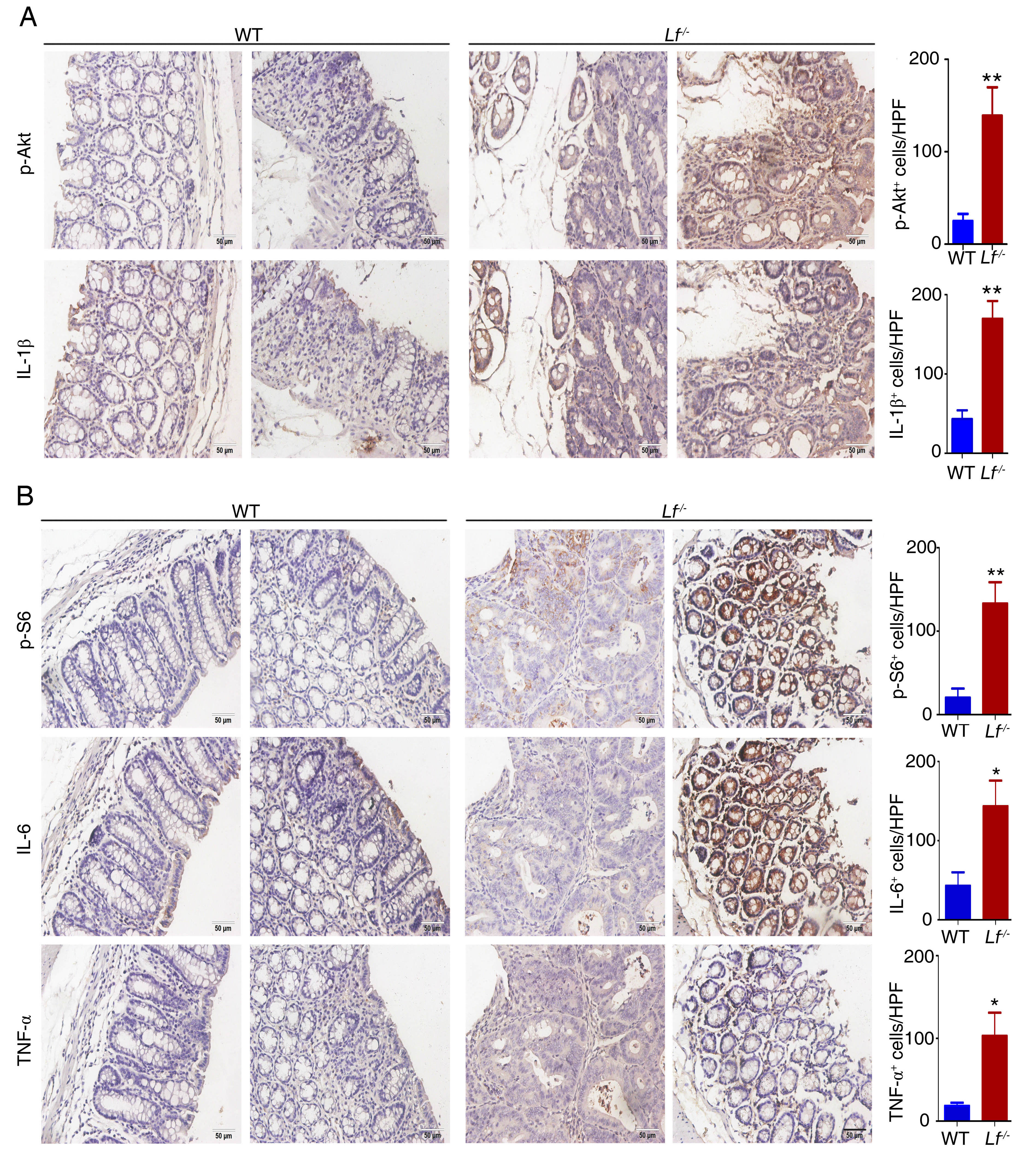

Supplement: Figure S1 — The association between the effect of cytokines abnormalities and AKT activation and mTOR deregulation. The colons were removed from AOM-DSS–treated mice after 18 weeks. (A) Immunohistochemistry analysis of serial sections of p-AKT and IL-1β expression levels in colon tissues. (B) Immunohistochemistry analysis of serial sections of p-S6, IL-6 and TNF-α expression levels in colon tissues. Cells that stained positive were counted per HPF (40× objective). *P<0.05, **P<0.01 versus WT mice. Scale bar is 50 µm. Each value represents the mean ± SD (n = 10 mice/group). (JPG) [file pone.0103298.s001.jpg]
