# Supplementary material for: Lactoferrin Deficiency Promotes Colitis-Associated Colorectal Dysplasia in Mice
Source: PLoS One. 2014 Jul 24;9(7):e103298. doi: 10.1371/journal.pone.0103298 (PMC4110006; doi:10.1371/journal.pone.0103298)
Supplement: Table S1 — Primers used for identification of mouse genotypes by PCR. (PDF) [file pone.0103298.s002.pdf]

Table S1. Primers used for identification of mouse genotypes by PCR

Name	Sequence of primers
P1	5’ GGTGCTTACTGCTCACATCG 3’
P2	5’ AAGCTGCCATGGCAAGGAGA 3’
P3	5’ GAGGCCCTTGGACTCTGTCTA 3’
P4	5’ TCTCAGACACCTCAAGGCTC 3’
Lf1	5’ TGCCATGACTCTTGATGGTGG 3’
Lf2	5’ CGTAGACTTCAGCTGCCACA 3’
Gadph-F	5’ TCTGACGTGCCGCCTGGAGA 3’
Gadph-R	5’ CAGCCCCGGCATCGAAGGTG 3’
