# Supplementary material for: Lactoferrin Deficiency Promotes Colitis-Associated Colorectal Dysplasia in Mice
Source: PLoS One. 2014 Jul 24;9(7):e103298. doi: 10.1371/journal.pone.0103298 (PMC4110006; doi:10.1371/journal.pone.0103298)
Supplement: Table S2 — Primers used for detection of NF-κB target genes by qPCR. (PDF) [file pone.0103298.s003.pdf]

Table S2. Primers used for detection of NF-κB target genes by qPCR

	Forward primers	Reversed primers
Il-1β	AGCCTCGTGCTGTCGGACCC	TCCAGCTGCAGGGTGGGTGT
Il-6	CCTCTCTGCAAGAGACTTCCATCCA	AGCCTCCGACTTGTGAAGTGGT
Cxcl1	GCCACCCGCTCGCTTCTCTG	CAAGGCAAGCCTCGCGACCA
Cox-2	GCTGTACAAGCAGTGGCAAA	CCCCAAAGATAGCATCTGGA
Mmp9	TGGTGTGCCCTGGAACTCA	TGGAAACTCACACGCCAGAAG
Ifn-γ	ACTGGCAAAAGGATGGTGAC	GACCTGTGGGTTGTTGACCT
Tnf-α	AGGGGCCACCACGCTCTTCT	CATGCCGTTGGCCAGGAGGG
Mcp-1	TCTGGGCCTGCTGTTCACA	CCTACTCATTGGGATCATCTTGCT
